# The *C. elegans gba-3* gene encodes a glucocerebrosidase that exacerbates α-synuclein-mediated impairments in deletion mutants

**DOI:** 10.1186/s40035-024-00463-4

**Published:** 2025-02-13

**Authors:** Ning Liu, Rongzhen Li, Xiaobing Huang, Merja Lakso, Garry Wong

**Affiliations:** 1 Faculty of Health Sciences, University of Macau, Macau S.A.R., 999078 China; 2 College of Pharmacy, Shenzhen Technology University, Shenzhen, 518000 China

Glucocerebrosidases (GCases) catalyze the hydrolysis of β-glucoceremides and β-glucosphingosines to produce glucose and ceramide or sphingosine, respectively. In humans, GCase is encoded by *GBA1* which is widely and abundantly expressed and localized in lysosomal membranes, while *GBA2* encodes a microsomal-β-glucosidase [1]. Loss-of-function mutations in *GBA1* result in accumulation of glucoceramides or glucosphingosines and underlie Gaucher’s disease (GD), a lysosomal storage disease characterized by anemia, enlarged spleen and liver, and skeletal disorders [2, 3]. The most common *GBA1* N370S mutant retains some residual activity while homozygosity results in a range of outcomes from mild symptoms to severe disease [4]. Specific *GBA1* mutations are the highest known genetic risk factors (odds ratio > 5) for Parkinson’s disease (PD) [5]. Nonetheless, knowledge of how GCase functional loss can increase PD risk is limited due to the lack of animal models [6]. In this study, we identified the gene encoding GCase in *C. elegans* via genetic and enzymatic activity analysis and transgenic overexpression. Interactions between GCase function and α-synuclein (α-syn) were investigated to gain further insight into how *GBA1* variations account for the largest risk factors for PD. Methods, strains, and primers are provided in Additional File 1: Table S1 and Table S2.

Gene nomenclature assignments predicted 4 GCase genes, *gba-1* to *gba-4,* with high identity to the human *hGBA1*. The genomic structures are shown **(**Fig. 1a**).** To verify that *C. elegans* has GCase activity, we assayed wild-type (WT) animals using the fluorogenic substrate 4-methylumbelliferyl-beta-D-glucopyranoside (MUB-Glc). WT animals had GCase activity comparable to HeLa cell extracts (0.69 ± 0.02 of WT) and the activity could be partially inhibited by the GBA1 inhibitor conduritol B epoxide (0.75 ± 0.03 of WT) **(**Fig. 1b**).** To determine which of the 4 *gba* genes encodes a GCase, we obtained deletion mutants and assayed their enzymatic activity. Only *gba-3*-deletion mutants showed a loss of activity (− 0.09 ± 0.01 of WT), while the other mutants retained activity similar to WT, strongly suggesting that *gba-3* encodes a GCase **(**Fig. 1b**).** Next, the *gba-3*-deletion animals were microinjected with either the *gba-3* or *hGBA1* cDNA constructs, and they showed recovery of GCase activity (0.31 ± 0.03 and 0.26 ± 0.03 of WT, respectively). Moreover, microinjection of the same constructs to WT animals increased GCase activity (1.39 ± 0.11 and 1.27 ± 0.06 of WT, respectively) **(**Fig. 1c**).** These results firmly demonstrate that *gba-3* is a *C. elegans* GCase-encoding gene.

**Fig. 1:**
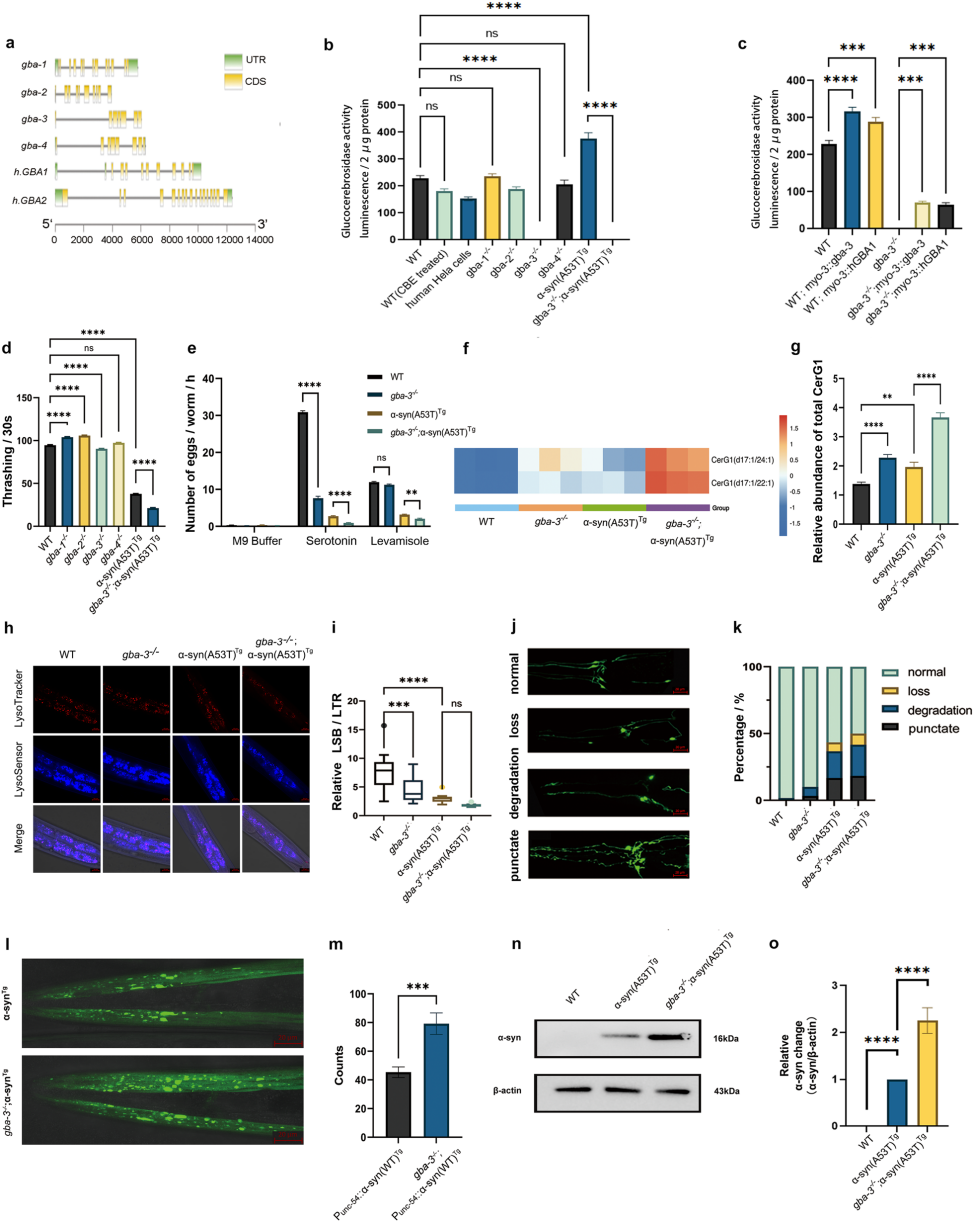
Phenotypic analysis of WT, *gba-3*^*−/−*^, α-syn(A53T)^Tg^, and *gba-3*^*−/−*^;α-syn(A53T)^Tg^ crosses. **a** Gene structures of *C. elegans gba* family members and human *GBA1* and *GBA2* genes. **b, c** Glucocerebrosidase enzymatic activity in relative fluorescence units (RFU). CBE, Conduritol-β-epoxide. **d, e** Locomotion (mean ± SEM, *n* = 30) and egg laying assays (mean ± SEM, *n* = 72)**. f, g** Heatmap and quantitation of glucosyl ceramides. **h, i** Confocal images and quantitation of Lysosensor Blue (LSB)-to-Lysotracker Red (LTR) staining ratio (*n* = 30). **j, k** Dopaminergic neuron morphology and quantitation. At least 30 animals were used for each strain. **l, m** Confocal imaging and quantitation of α-syn aggregates in *unc-54p::α-syn(WT)::YFP* (NL5901) and *unc-54p::α-syn(WT)::YFP* (NL5901);*gba-3*^−/−^ crosses (*n* = 30). **n, o** Western blot and quantitation of α-syn protein levels in WT and transgenic animals (*n* = 3). ****P < 0.0001, ***P < 0.001, **P < 0.01, *P < 0.05; ns, no significance using one-way ANOVA with Sidak’s multiple comparisons post-hoc test. For data that were not normally distributed, Kruskal-Wallace was used. Two-tailed *t*-test was used for metabolite comparisons

To investigate the interactions between *gba-3* and α-syn, the main pathogenic protein in PD, we crossed *gba-3* deletion mutants to a previously established *C. elegans* PD model that pan-neuronally overexpresses human α-syn(A53T) [7]. This PD model had higher GCase activity than WT (1.64 ± 0.04), but crossing with the *gba-3* deletion mutants led to loss of enzymatic activity (Fig. 1b). To characterize the effects of GCase activity loss on PD phenotypes, we assayed movement. Compared to WT (94.6 ± 0.6), as well as *gba-1-* (104.0 ± 0.6), *gba-2-* (105.8 ± 0.6), *gba-3-* (90.3 ± 0.5) and *gba-4-*deletion (97.3 ± 0.4) mutants, the α-syn(A53T)^Tg^ animals had decreased thrashing behavior (37.8 ± 0.4), and *gba-3* deletion further decreased the thrashing behavior of the PD model (21.1 ± 0.7) (Fig. 1d). We also observed significantly weaker egg laying in response to serotonin stimulation in *gba-3* deletion mutants (7.63 ± 0.5) compared to WT (30.9 ± 0.4). The response was further weakened in the PD model (α-syn(A53T)^Tg^, 2.75 ± 0.2; *gba-3*^*−/−*^;α-syn(A53T)^Tg^, 0.90 ± 0.1) **(**Fig. 1e**).** This phenotypic deficit was not observed with the cholinergic agonist levamisole where *gba-3* mutants responded similar to WT and *gba-3* deletion did not significantly alter the weak response of the PD model **(**Fig. 1e**)**. Thus, at least 2 different assays showed exacerbation of phenotypic deficits by *gba-3* mutants on the PD model.

Next, we wanted to determine if GCase activity loss could lead to accumulation of unreacted substrate, as previous studies suggested involvement of lipid pathway dysregulation in mediating PD pathology [8]. Using a targeted metabolomics approach, we observed increases in glucosylceramides CerG1(d17:1/24:1) and CerG1(d17:1/22:1), which are putative substrates for GCase (Fig. 1f). We also observed accumulation of the same CerG1 substrates in the human α-syn(A53T)-overexpressing animals, and *gba-3* deletion further exerted an additive effect (WT, 1.383 ± 0.03631; *gba-3*^−/−^, 2.282 ± 0.06307; α-syn(A53T)^Tg^, 1.966 ± 0.09361; *gba-3*^−/−^;α-syn(A53T)^Tg^, 3.664 ± 0.09231) (Fig. 1g). These results suggest that the disturbed fatty acid homeostasis in PD may be further exacerbated by *gba-3* deletion.

Lysosomes are implicated in the disease progression of both GD and PD [9]. We explored the status of the lysosomes using lysotracker dyes and observed decreased functional capacity (LysoSensor blue/LysoTracker red) in *gba-3*^−/−^ mutants and α-syn(A53T)-overexpressing animals (Fig. 1h). However, the lysosomal impairment in α-syn(A53T)^Tg^ animals was not significantly changed by *gba-3* deletion (Fig. 1i). The low level of lysosomal function in the PD model may have prevented a more severe impairment.

We next investigated the morphology of dopaminergic neurons (Fig. 1j). The percentages of normal morphology in WT, *gba-3*^−/−^, α-syn(A53T)^Tg^, and *gba-3*^−/−^;α-syn(A53T)^Tg^ were 98.3% ± 0.017%, 91.7% ± 0.030%, 56.7% ± 0.033%, and 50% ± 0.029%, respectively **(**Fig. 1k**)**. Confocal microscopy indicated that the *gba-3*^−/−^ animals had slightly more abnormalities than WT, and *gba-3* deletion added only slightly to the percentage of impairment in α-syn(A53T)^Tg^ (Fig. 1k). This suggests that *gba-3* function may only slightly contribute to dopaminergic neuron degeneration.

Finally, we wanted to determine the α-syn protein level to test if *gba-3* deletion could contribute to increased risk of PD in this manner. Using a transgenic line that overexpresses α-syn fused to YFP in muscle cells [10], we observed increased α-syn aggregation in *gba-3*-deletion mutant animals (control 45.3 ± 3.7 vs. *gba-3 *^*−/−*^ 79.2 ± 7.5) (Fig. 1l, m). Western blots showed that the total protein level of α-syn increased significantly in *gba-3*-deletion mutants (fold change 2.109 ± 0.227) (Fig. 1n, o**;** uncropped images available in Additional File 2). These results indicate that loss of GCase function could further increase α-syn protein levels in vivo. This increase suggests a sequence of events that ultimately lead to neurodegeneration.

In this study, by establishing *gba-3* as a *C. elegans* GCase-encoding gene, we could genetically cross loss-of- function mutants to an α-syn(A53T)-overexpressing line and explore their interactions. We observed exacerbation of movement deficits and serotonergic signaling. However, we did not observe any worsening of lysosomal function, although it was already at a low level, but did observe increased accumulation of CerG1 substrates that were already higher in the PD transgenic animal model. A significant finding is that loss of GCase activity led to increased α-syn protein levels. As α-syn is a key protein in Lewy bodies and central to PD pathology, this may lead to an increased risk. Importantly, we observed only modest changes to dopaminergic neuron degeneration in loss-of-function mutants, suggesting that the effects of GCase activity loss may occur early, perhaps before neurodegeneration. Thus, translational approaches to enhancing GCase activity should be used at an early stage to exert beneficial effects. We do not know if *gba-3* loss-of-function mutation may affect memory or other neurodegenerative processes, therefore, future studies are required to address this in a rigorous manner. Finally, our investigation provides two new animal models for studying GD or PD, respectively, which may further aid in the understanding of these neurodegenerative disorders.

## Supplementary Information

Additional file 1. **Methods**. **Table S1**. Strains used in this study. **Table S2**. Primers used in this study.

Additional file 2. Uncropped images of Western blots.

## Abbreviations

GCase: Glucocerebrosidase
GD: Gaucher’s disease
PD: Parkinson’s disease
WT: Wildtype

## Declarations

### Availability of data and materials

The datasets supporting the conclusions of this article are included within the article and its additional files.

## References

[1] Boot RG, Verhoek M, Donker-Koopman W, Strijland A, van Marle J, Overkleeft HS, et al. Identification of the non-lysosomal glucosylceramidase as beta-glucosidase 2. J Biol Chem. 2007;282:1305–12. 17105727 10.1074/jbc.M610544200

[2] Nilsson O, Grabowski GA, Ludman MD, Desnick RJ, Svennerholm L. Glycosphingolipid studies of visceral tissues and brain from type 1 Gaucher disease variants. Clin Genet. 1985;27:443–50. 3924448 10.1111/j.1399-0004.1985.tb00229.x

[3] Stirnemann J, Belmatoug N, Camou F, Serratrice C, Froissart R, Caillaud C, et al. A review of gaucher disease pathophysiology, clinical presentation and treatments. Int J Mol Sci. 2017;18:441. 28218669 10.3390/ijms18020441 PMC5343975

[4] Fairley C, Zimran A, Phillips M, Cizmarik M, Yee J, Weinreb N, et al. Phenotypic heterogeneity of N370S homozygotes with type I Gaucher disease: an analysis of 798 patients from the ICGG Gaucher registry. J Inherit Metab Dis. 2008;31:738–44. 18979180 10.1007/s10545-008-0868-z

[5] Zhang X, Wu H, Tang B, Guo J. Clinical, mechanistic, biomarker, and therapeutic advances in *GBA1*-associated Parkinson’s disease. Transl Neurodegener. 2024;13:48. 39267121 10.1186/s40035-024-00437-6 PMC11391654

[6] Koprivica V, Stone DL, Park JK, Callahan M, Frisch A, Cohen IJ. Analysis and classification of 304 mutant alleles in patients with type 1 and type 3 Gaucher disease. Am J Hum Genet. 2000;66:1777–86. 10796875 10.1086/302925 PMC1378059

[7] Lakso M, Vartiainen S, Moilanen AM, Sirvio J, Thomas JH, Nass R, et al. Dopaminergic neuronal loss and motor deficits in Caenorhabditis elegans overexpressing human alpha-synuclein. J Neurochem. 2003;86:165–72. 12807436 10.1046/j.1471-4159.2003.01809.x

[8] Galper J, Dean NJ, Pickford R, Lewis SJG, Halliday GM, Kim WS, et al. Lipid pathway dysfunction is prevalent in patients with Parkinson’s disease. Brain. 2022;145:3472–87. 35551349 10.1093/brain/awac176 PMC9586542

[9] Wong YC, Krainc D. Lysosomal trafficking defects link Parkinson’s disease with Gaucher’s disease. Mov Disord. 2016;31:1610–8. 27619775 10.1002/mds.26802 PMC5957289

[10] van Ham TJ, Thijssen KL, Breitling R, Hofstra RM, Plasterk RH, Nollen EAC. elegans model identifies genetic modifiers of alpha-synuclein inclusion formation during aging. PLoS Genet. 2008;4: e1000027. 18369446 10.1371/journal.pgen.1000027 PMC2265412

