# Supplementary material for: The C. elegans gba-3 gene encodes a glucocerebrosidase that exacerbates α-synuclein-mediated impairments in deletion mutants
Source: Transl Neurodegener. 2025 Feb 13;14:9. doi: 10.1186/s40035-024-00463-4 (PMC11823175; doi:10.1186/s40035-024-00463-4)
Supplement: Supplementary file 1 — Additional file 1. Methods. Table S1. Strains used in this study. Table S2. Primers used in this study. [file 40035_2024_463_MOESM1_ESM.docx]

**Additional File 1: Supplementary Materials**

**Methods**

***C. elegans* maintenance and genetics**

Nematode growing medium (NGM) plates were used to cultivate *C. elegans* according to standard methods. *Escherichia coli* strain OP50 served as the food source. Genetic crosses were obtained using standard methods. N2 served as the wildtype (WT) strain. N2, NL5901, and CB1111 were obtained from the Caenorhabditis elegans center. Mutant strains *gba-1*^-/-^ (tm3816)*, gba-2*^-/-^ (tm4623), *gba-3*^-/-^ (tm3302), *gba-4*^-/-^ (tm3349) were obtained from the National BioResource Project (Tokyo, Japan). Integrated strain UM0009 *Is[dat-1p::GFP];* and UM0010 *Is[dat-1p::GFP; aex-3p::α-syn(A53T))* were previously published [7]. LN1 was generated by crossing the PD model strain (Is[*dat-1p::GFP; aex-3p::α-syn(A53T*))] and tm3302(*gba-3*^-/-^). LN2 was generated by crossing NL5901 *(Is[unc-54p:: α-synuclein::YFP*]) and tm3302(*gba-3*^-/-^). LN3 was generated by crossing (Is[*dat-1p::GFP*]) and tm3302 (*gba-3*^-/-^). Strains and their sources are listed below. Strains were crossed to homozygosity and confirmed with the PCR primers listed below.

**Construction of overexpression plasmids**

In this project, 3 plasmids were constructed. The h.GBA1 cDNA template was obtained from pCMV-SPORT6-GBA, purchased from MiaoLing Bio, China. The gene was amplified with primer set phGBA1-F/R, then purified. Each PCR amplicon was inserted by into the vector HJ35 which was linearized by PCR with primer set pVhGBA1-F/R by homologous recombination. Plasmid pmyo-3::hGBA1(L444P) was generated by amplification with primer set phGBA1mut-F/R. Plasmid prab-3::*gba-3* was generated by Yunzhou Company (Guangzhou, China). After sequencing for verification, the created plasmids were mixed with pdat1::GFP plasmid and microinjected into worm strains N2 (WT) and tm3302 (*gba-3*^-/-^) at 100ng/µl. At least 10 independent transgenic lines were generated for each construction. Strains with higher heritability of the transgenic marker gene were selected for assays. Primers used are listed in below.

**GCase enzymatic assay**

Fluorogenic enzyme assay was employed to test the GCase activity level. 4-methylumbelliferyl-beta-D-glucopyranoside (MUB-Glc, CAS: 18997-57-4, Sigma Aldrich) performed as the substrate. Upon the hydrolysis of MUB-Glc, methyllumbelliferone can be excited at 365 nm and emits at 440 nm. For sample preparation, animals were collected and proteins were extracted and quantified by Pierce™ BCA protein assay kit (Thermo Fisher Scientific). Then substrate was solubilized at a final concentration of 1.5 mM in Mcllvaine Buffer containing 0.1% Triton X-100. A 20µL solution was added into each well of a 96-well plate. A concentration gradient was produced by adding different volumes of protein and solvent, to reach the total volume is 30 µL. The experiment was performed with total protein amounts of 0, 2 µg, 4 µg, 6µg, and 8 µg. The reaction mixtures were gently mixed and incubated at 37°C for 1 h. The reaction was stopped by transferring 10 µL of the mixture into wells with 190 µL of 0.25 M glycine, pH 10.7. The fluorescence was detected by PerkinElmer Victor X3 Microplate Reader. The data was expressed as relative change to WT.

**Egg-laying assay**

Age-synchronized animals were cultivated at 20°C until they grew to day 2 adults. Then they were transferred to 96 well plates. Hermaphrodites were then randomly selected and transferred into 96-well plates. Two worms were picked in each well with 100ul of 5mg/ml serotonin, or 10mg/ml levamisole, or M9 buffer. After an hour, the number of eggs in each well was counted under the microscope.

**Thrashing assay**

For thrashing experiments, worms were cultured at 20°C until L4 stage. Then the worms were randomly picked and placed in liquid M9 buffer. After a recovery period of 30 seconds, their frequency of thrashing movements were recorded for 30 seconds. A total of 30 worms were counted in each experiment, and three biological replicates were performed.

**Lysosome staining**

Age-synchronized animals were cultivated at 20°C until they reached Day1 adults, then collected with M9 buffer and washed for 3 times. Staining was carried out in M9 buffer containing 60µM LysoTracker red [Invitrogen, Waltham, MA, USA] and 60µM LysoSensor blue [Invitrogen] for 1 h at room temperature in the dark. Worms then were transferred onto NGM plates seeded with OP50 and allowed to recover for 1 hour, after which they were picked up and paralyzed by 15mM sodium azide and mounted onto slides. Images were taken by a Zeiss confocal microscope LSM710 using the 63X objective using an Argon laser (Lasos Model LGN3001, Lasos Lasertechnik GmbH), with Alexa (LysoTracker) and DAPI (LysoSensor) filters, 6.30 µsec exposure, and image size of 1024*1024 pixels. Florescence intensity was quantified by Image J. At least 30 worms were scored in each experiment.

**Visualization of dopaminergic neurons**

At least 30 animals at Day1 adult stage were picked and paralyzed with 15mM sodium azide and mounted onto slides. The dopaminergic neurons were observed and imaged by a Zeiss confocal microscope LSM710 using the GFP filter. The conditions of the dopaminergic neurons were defined as described. At least 30 animals were scored in each group.

**Immunoblotting**

Animals were cultivated at 20°C until they reached L4 stage, then collected with M9 and washed for 3 times, and then dissolved in Tris-Urea-SDS buffer (1× TBS, 5% SDS, 8 M Urea, 50 mM dithiothreitol) . Animals were sonicated for 30 minutes (Bioruptor® Plus sonication device) to lyse the cells and extract the protein, followed by centrifuging at 14000 rpm for 30 min to isolate the protein lysate. Total protein concentration was determined by Pierce™ BCA protein assay kit (Thermo Fisher Scientific). A total of 20µg of proteins were loaded on to SDS-page gel and electro transferred to PVDF membrane (Merck, China). Primary anitibody anti-α-syn (1:1000, Thermo/ PA5-85343) and anti-β-actin (1:1000, Santa Cruz/ sc-47778) was incubated together at 4°C overnight. The membrane was washed for 3 times by shaking with PBST for 15 min. For detection, secondary antibody Peroxidase AffiniPure Goat Anti-Rabbit IgG (1:5000, Jackson ImmunoResearch/111035144) and Peroxidase AffiniPure Goat Anti-Mouse IgG (1:5000, Jackson ImmunoResearch/115035146) were used by incubation for 1 h at room temperature. Immunocomplexes were exposed to ECL substrate (Bio-Rad, Catalog # 170-5061) and visualized by the ChemiDoc MP Imaging Systems (Bio-Rad). Quantitation of ratio of bands was calculated based upon subtracting background from the blot, normalizing to β-actin, then subtracting any remaining signal from WT.

**Lipidomic analysis with Liquid Chromatography Mass Spectrometry (LC-MS)**

For the sample preparation, *C. elegans* were collected, freeze-dried, and then stored at -80°C. Samples were thawed at room temperature, mixed with 300µl precooling methanol (A452-4, Thermo Fisher Scientific) and transferred into a 1.5 mL Eppendorf tube. Samples were vortexed for 1 min, and then 800µl MTBE ( E127-4,, Thermo Fisher Scientific) was added and vortexed for 1 min to mix. Next, 20 µl of DI water was added and and vortexed. The mixtures were extracted by ultrasonication for 10 min in an ice-water bath, then ultrasonication for 20 min in an ice-water bath then placed at room temperature for 30 min. The sample was then centrifuged at 4°C (13000 rpm) for 15 min to obtain the upper organic phase. The mixed supernatant was dried under a nitrogen stream and redissolved in isopropanol: acetonitrile: water (v:v:v = 14:5:1), extracted by ultrasonication for 5 min in an ice-water bath, vortexed for 1 min, and then centrifuged at 4°C (13000 rpm) for 15 min. Supernatant was taken for the later UPLC-MS/MS analysis. QC samples were prepared by mixing an aliquot of all samples as a pooled sample.

For UPLC_MS/MS, an ACQUITY UPLC I-Class plus（Waters Corporation,Milford, USA）fitted with Q-Exactive mass spectrometer equipped with heated electrospray ionization(ESI) source (Thermo Fisher Scientific, Waltham, MA, USA) was used to analyze the metabolic profiling in both ESI positive and ESI negative ion modes. An ACQUITY UPLC BEH C8 （100 mm×2.1 mm, 1.7 um） were employed in both positive and negative modes. The binary gradient elution system consisted of (A) acetonitrile: water (60:40, v: v, containing 10mmol/L ammonium formate) and (B) acetonitrile: isopropanol (10:90, v: v, containing 10mmol/L ammonium formate) and separation was achieved using the following gradient:0 min, 32% B; 1.5 min, 32% B; 15.5 min, 85% B; 15.6 min, 97% B; 18.0 min, 97% B; 18.1 min, 32% B, 20 min, 32% B. The flow rate was 0.26 mL/min and column temperature was 55°C. The injection volume was 3 uL. Positive: Sheath Gas Flow rate 45 arb, Aux Gas Flow Rate 10 arb, spray voltage 3.5 KV, Capillary Temp 320°C, S-F Level 50%. MS1 scan ranges: 120-1800. Negative: Heater Temp 300°C, Sheath Gas Flow rate 45 arb, ALens Rux Gas Flow Rate 15 arb, Sweep Gas Flow Rate 1 arb, spray voltage 3.1 KV, Capillary Temp 300°C, S-Lens RF Level 50%. MS1 scan ranges: 150-1500.

For data processing and analysis, the initial Q Exactive LC-MS/MS data in .raw format underwent processing using LipidSearch software for MSn. The identification of lipid molecular structures was based on the parent ions and multi-stage mass spectrometry data specific to each sample. The results were then aligned within a defined retention time range and consolidated into a comprehensive report to organize the original data matrix. Normalization was applied to all peak signals in each sample. Subsequently, any peaks with missing values (ion intensity = 0) exceeding 50% within groups were eliminated, and zero values were replaced with half of the minimum value. A combined data matrix was created by merging positive and negative ion data sets. To distinguish metabolites that exhibited differences between groups, Orthogonal Partial Least-Squares-Discriminant Analysis (OPLS-DA) and Partial Least-Squares-Discriminant Analysis (PLS-DA) techniques were employed. In order to prevent overfitting, model quality evaluation involved 7-fold cross-validation as well as 200 Response Permutation Testing (RPT). Variable Importance of Projection (VIP) values derived from the OPLS-DA model served as an indicator for ranking the overall contribution of each variable towards group discrimination. Furthermore, a two-tailed Student's T-test was conducted to verify statistical significance among differentially expressed metabolites between groups. Differential metabolites meeting criteria such as VIP values ≥ 1.0 and p-values ≤ 0.05 were selected.

**Statistical analysis**

One-way ANOVA, Sidak’s multiple comparisons test was used to compare strains based on one factor. We also applied the Shapiro-Wilk test to determine if the data was normally distributed. If it was not, we applied the Kruskal-Wallis test. Student’s t-test was also used as appropriate. Data analysis was performed by GraphPad Prism (GraphPad Software, San Diego, CA,USA) and shown as mean ± SEM unless otherwise indicated.

**Table S1. Strains used in this study.**

| **Strain** | **Genotype** | **Transgene Expression** | **Source** |
| --- | --- | --- | --- |
| N2 | Wild type | - | CGC |
| UM0009 | *dat-1p::GFP* | Dopaminergic neurons | [6] |
| UM0010 | *dat-1p::GFP;aex-3p::α-syn(A53T)* | Dopaminergic neurons, pan-neuronal | [6] |
| NL5901 | *unc-54p::α-syn::YFP* | Body-wall muscle | CGC |
| tm3816 | *gba-1 ^-/-^* | - | NBRP |
| tm4623 | *gba-2 ^-/-^* | - | NBRP |
| tm3302 | *gba-3 ^-/-^* | - | NBRP |
| tm3349 | *gba-4 ^-/-^* | - | NBRP |
| LN1 | *gba-3 ^-/-^;dat-1p::GFP;aex-3p::α-syn(A53T)* | Dopaminergic neurons, pan-neuronal | This project |
| LN2 | *gba-3 ^-/-^;dat-1p::GFP;aex-3p::α-syn(A53T)* | Dopaminergic neurons, pan-neuronal | This project |
| LN3 | *gba-3 ^-/-^;unc-54p::α-syn::YFP* | Body-wall muscle | This project |
| LN4 | *myo-3p::hGBA1(L444P)* | Body-wall muscle | This project |
| LN5 | *gba-3^-/-^ ;myo-3p::hGBA1(L444P)* | Body-wall muscle | This project |
| LN6 | *rab-4p::gba-3* | Pan-neuronal | This project |
| LN7 | *gba-3 ^-/-^; rab-4p::gba-3* | Pan-neuronal | This project |

Abbreviations: CGC, Caenorhabditis Genetics Center; NBRP, National BioResources Project.

**Table S2. Primers used in this study.**

**
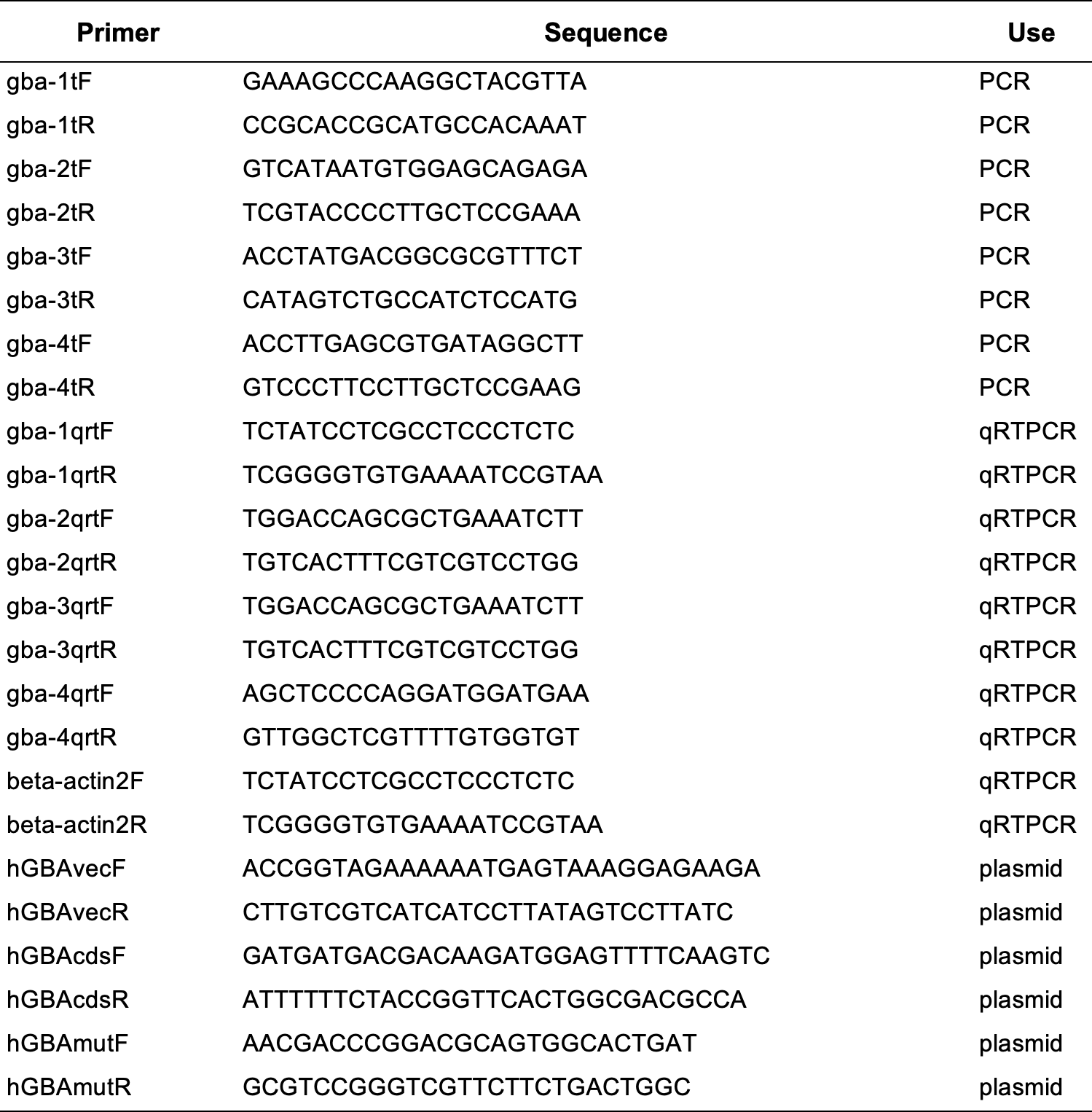
**
