# Supplementary figures and images for: The C. elegans gba-3 gene encodes a glucocerebrosidase that exacerbates α-synuclein-mediated impairments in deletion mutants
Source: Transl Neurodegener. 2025 Feb 13;14:9. doi: 10.1186/s40035-024-00463-4 (PMC11823175; doi:10.1186/s40035-024-00463-4)

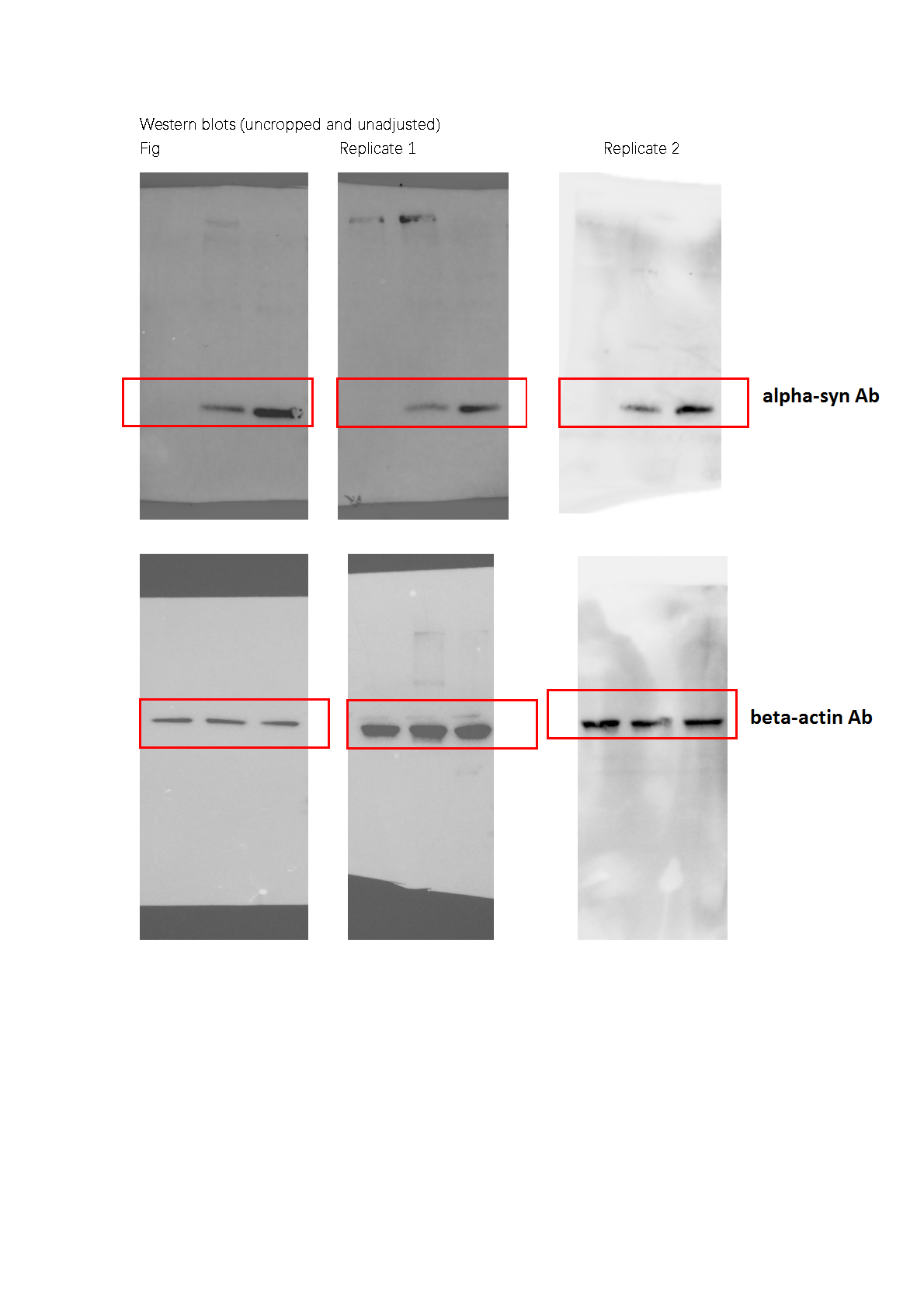

Supplement: Supplementary file 2 — Additional file 2. Uncropped images of Western blots. [file 40035_2024_463_MOESM2_ESM.tif]
